# Association of chronotype and depression symptoms in Chinese infertile population undergoing assisted reproductive technology

**DOI:** 10.3389/fpsyg.2025.1423418

**Published:** 2025-06-13

**Authors:** Fei Jiang, Yuedi Jia, Xiaohuan Song, Mengli Zhu, Xin Wang, Guiying Luo, Jieyu Wang, Qianhua Xu, Danni Wang

**Affiliations:** ^1^Department of Nutrition, School of Public Health, Anhui Medical University, Hefei, China; ^2^Department of Health Promotion and Behavioral Sciences, School of Public Health, Anhui Medical University, Hefei, China; ^3^Department of Clinical Nutrition, The Yancheng School of Clinical Medicine of Nanjing Medical University, Yancheng Third People’s Hospital, Yancheng, China; ^4^Department of Obstetrics and Gynecology, Reproductive Medicine Center, The First Affiliated Hospital of Anhui Medical University, Hefei, China

**Keywords:** chronotype, depression symptoms, infertility, assisted reproductive technology, cross-sectional study

## Abstract

**Objective:**

To evaluate the association between chronotype and depression symptoms in a group of individuals experiencing infertility and undergoing assisted reproductive technologies.

**Methods:**

This cross-sectional study evaluated the eligibility of 1,022 infertile patients who underwent ovulation induction treatment at the First Affiliated Hospital of Anhui Medical University Reproductive Medicine Center in China between August and October 2022. We obtained socio-demographic information by inviting patients who were experiencing infertility to fill out questionnaires. Furthermore, we evaluated the participants’ chronotypes using the reduced Morningness-Eveningness Questionnaire (rMEQ). Depression symptoms were evaluated using the validated Patient Health Questionnaire-9 (PHQ-9) and were expressed as PHQ-9 scores.

**Results:**

The participants were classified into evening, intermediate, and morning chronotypes, accounting for 9, 68, and 22% of the total, respectively. There were considerable variations in levels of depression symptoms based on chronotype. Patients with morning chronotype had lower odds of depression symptoms (odds ratios = 0.32, 95% confidence intervals: 0.18–0.57), as did those with intermediate chronotype (odds ratios = 0.47, 95% confidence intervals: 0.28–0.77), compared to individuals with evening chronotype. Furthermore, there were no significant partner effects of chronotype on depression symptoms in male and female dyads (*p* > 0.05 for both).

**Conclusion:**

The results indicated a significant association between individuals who have morning and intermediate chronotypes and a reduced likelihood of experiencing depressed symptoms. Further studies are required to assess the partner effect of chronotypes on depression symptoms.

## Introduction

Infertility is a medical condition that denotes the inability of a person or couple to achieve a clinical pregnancy even after 12 or more months of regular unprotected sexual intercourse (Zegers-Hochschild et al., 2017). According to epidemiological surveys, this condition is becoming increasingly common, affecting 186 million couples worldwide (Inhorn and Patrizio, 2015). Younger individuals are also affected, with a prevalence of 10–15% (Tamrakar and Bastakoti, 2019). In China, infertility affects 25% of couples of reproductive age (Zhou et al., 2018), making it the third most significant health issue after cardiovascular diseases and tumors (Agarwal et al., 2020). The implementation of China’s fertility policy exerts significant pressure on infertile individuals from society, family, and the treatment process. This pressure arises from their long-term inability to have children naturally, leading to considerable psychological burden and social–emotional distress. Consequently, infertility patients experience a significant impact on their physical and mental health (Cocchiaro et al., 2020; Kamboj et al., 2023).

Depression is a common mental disorder characterized by a continuous state of poor mood. It affects roughly 322 million individuals, about 4.4% of the global population (Friedrich, 2017). Depression is the second most significant contributor to disability globally (Refisch et al., 2023). There is strong evidence indicating a link between depression and metabolic syndromes (Ferriani et al., 2023), such as obesity (Ramzi et al., 2023), hypertension (Shah et al., 2023), and immuno-metabolic dysregulation (Toenders et al., 2022), which can lead to negative long-term health outcomes. Depression also has an impact on secondary infertility(Sun et al., 2024), and the outcomes of assisted reproductive technology (ART) treatments (Purewal et al., 2018; Vo et al., 2019; Aimagambetova et al., 2020). This includes a reduction in the chances of getting pregnant and having a live birth (Cesta et al., 2016; Evans-Hoeker et al., 2018), as well as a decrease in the success rates of ART treatment cycles (Sejbaek et al., 2013). Depression also increases the risk of having a low birth weight infant (Lang et al., 2019). Infertile patients are more likely to experience depression due to the prolonged duration of infertility, numerous contributing factors, expensive treatment expenses, arduous treatment procedures, and the impact of social culture and traditional beliefs. According to reports, around 51.3% of women who were unable to conceive naturally experienced depression while undergoing *in vitro* fertilization-embryo transfer (IVF-ET) treatment (Zhou et al., 2023). Women receiving infertility therapy have a higher incidence of depression compared to women who have routine medical checks or conceive naturally (Wang et al., 2024). Depression is linked to various factors, including both social risk factors and individual-level lifestyle factors, such as disruptions in circadian rhythm, which can significantly impact the development and progression of depression, as well as the overall severity of depression symptoms (Monteggia and Kavalali, 2012).

The collected evidence suggests that circadian rhythm disturbances are associated with many adverse health outcomes (Comas et al., 2023), such as obesity (Li et al., 2020), type 2 diabetes (Peng et al., 2022), cardiovascular diseases (Mentzelou et al., 2023), reproductive functions (Caetano et al., 2021), psychiatric disorders (Codoner-Franch et al., 2023), and cancer (Lotti et al., 2023). The chronotype is generally considered to be one of the manifestations of circadian rhythms. Differences in sleep–wake patterns across individuals can be used to determine distinct chronotypes, which reflect behavioral patterns and physiological processes that influence preference to engage in various lifestyle behaviors over the 24 h of the day (Roenneberg et al., 2007; Senesi et al., 2022), which can be divided into morning, intermediate, and evening chronotypes (Park et al., 2018). The morning chronotype is commonly defined by a tendency to sleep and wake up early and increased activity throughout the morning hours. Conversely, individuals with an evening chronotype tend to sleep, wake up late, and prefer working later in the day (Malin et al., 2024). Previous researchers showed that evening chronotype was a risk factor for mental health and that individuals with morning chronotype were at lower risk of developing emotional problems such as anxiety and depression compared to those with an evening chronotype (Antypa et al., 2016). With the increasing prevalence of infertility, researchers are increasingly focusing on the chronotype among infertile individuals. They have found that infertile populations tend to have poorer sleep quality and a preference for evening chronotype is more frequent as compared to those who are fertile (Ozcelik et al., 2023). Furthermore, Researchers showed that chronotype has an impact on the levels of testosterone secretion in infertile men and that evening chronotype may negatively affect sperm morphology in these individuals (Gica et al., 2023; Lu et al., 2024). However, Epidemiologic studies of chronotype and fertility treatment outcomes are relatively limited and not yet consensus, which may be related to differences in population or assessment of chronotype (Li et al., 2024). A prospective study showed that morning chronotype was associated with undesirable IVF-ET outcomes, including the lowest rates of clinical pregnancy and live birth and the highest rate of miscarriage (Liu et al., 2023). Another prospective study found that a mid-sleep time (MST) earlier than 2:21 a.m. or later than 3:00 a.m. was associated with significantly lower fertilization rates (Yao et al., 2022).

Indeed, it is imperative to analyze the influence of chronotypes on depression during infertility treatment. Infertility is a more specific reproductive disorder that can significantly affect the individual, their spouse, and possibly the entire family. Depression symptoms pose a significant issue in the reproductive therapy of patients with infertility, and it is crucial to investigate the variables that increase the risk to enhance overall health and well-being throughout their lives. To the best of our knowledge, there is a lack of clarity about the relationships between chronotype and depression symptoms during infertility treatment. The primary objective of this study was to investigate the impact of different sleep patterns on depression symptoms in individuals undergoing infertility treatment. The purpose of this study is twofold: firstly, to evaluate the impact of spouses’ chronotypes on their depression symptoms, and secondly, to examine how one partner’s chronotype affects the other partner’s depression symptoms.

## Materials and methods

### Participants

This cross-sectional study was conducted from August to October 2022 among infertile couples undergoing ovulation induction treatment at the Center for Reproductive Medicine, First Affiliated Hospital of Anhui Medical University (Hefei, Anhui, China). Participants who had a previous psychiatric illness, did not undergo embryo transfer, had other medical conditions such as hypertension, diabetes, or kidney disease, or chose to withdraw from the study were not included in the final sample (*n* = 177). For this study, baseline data were collected through a questionnaire, and the participants scanned a QR code using a mobile application to access the questionnaire and complete it online while being closely monitored by well-trained research personnel, with a final response rate of 85.7%. The questionnaire encompassed a range of topics, including demographic characteristics (age, sex, ethnicity, education level, income, occupation, and marital status), behavioral lifestyle (such as smoking or passive smoking, alcohol and coffee consumption, Sleep, psychological state, physical activity, and dietary habits), family and social relationships (such as social capital, family power and reproductive quality of life) and history of fertility treatment (such as parity, gravidity, history of preterm birth and abortion, infertility treatment timing and causes). Finally, 1,022 infertile patients were included in the final analysis. A post-hoc power analysis was conducted to evaluate the adequacy of the sample size for detecting the observed effect sizes. Based on a two-tailed *α* = 0.05 and 80% power, our final cohort of 1,022 participants provided >99% statistical power for the observed effects. The study protocol received approval from the Ethics Committee of Anhui Medical University (No. 20200961) based on the guidelines of the Declaration of Helsinki and international ethical standards. All invited participants have obtained a written informed consent form to participate in the study (Supplementary Figure S1).

### Chronotype assessment reliability and validity

The Morningness-Eveningness Questionnaire (MEQ) is the most often used scale for measuring circadian rhythms. The MEQ-19, first proposed by Horne and Ostberg (1976) is a specific version of this questionnaire. To efficiently assess the chronotypes, we employed the reduced Morningness-Eveningness Questionnaire (rMEQ), a subset derived from the MEQ-19 by Adan and Almirall (1992) using statistical modeling techniques and consisting of 5 items. The rMEQ is a valid and reliable scale for chronotype in many languages (Carciofo et al., 2012; Danielsson et al., 2019; Uygur et al., 2024). Despite the fact that validation in infertile individuals is yet more limited, the Cronbach’s alpha coefficient was 0.703, Split-half reliability was 0.612, and the significant correlation between the rMEQ scores and chronotype classifications (*r* = 0.824) in the present study. Also, exploratory factor analysis showed that all item factor loadings were found to meet the conventional threshold of >0.4 (range from 0.431to 0.792) (Giesinger et al., 2016), supporting the reliability and validity of the Chinese rMEQ. The widely adopted cut-off value from prior studies was used in this study (Caci et al., 2009). The chronotype of respondents was categorized into evening chronotype (4–11 score), intermediate chronotype (12–17 score), and morning chronotype (18–25 score) based on the rMEQ scores. While independent validation in our population was not performed, this approach was necessary to be aligned with existing literature to ensure comparability.

### Depression symptoms assessment

The participants were also instructed to provide their psychological status for the previous week using the Patient Health Questionnaire-9 (PHQ-9). The self-rating scale for depression disorder is a straightforward and efficient tool that is based on the diagnostic criteria of the American Psychological Association (Kroenke, 2021). Previous studies have demonstrated its reliability and validity (Yin et al., 2022), with Cronbach’s alpha coefficient of 0.910 in the present study. The assessment consists of nine items; the total score ranges from 0 to 27. A higher score indicates a greater severity of depression symptoms. In this study, in order to capture mild or subthreshold depressive symptoms, considering the sensitivity of the non-clinical population as well as the specificity of the infertility patients (Löwe et al., 2004; Coward et al., 2019). The total score was divided into a dichotomous variable. A score greater than 4 points indicates the presence of mild or more severe depression symptoms, while a score below 4 indicates the absence of depression symptoms.

### Covariates

All covariates were obtained from self-reported baseline questionnaires. We thoroughly searched the existing literature and employed a directed acyclic graph to identify potential confounding factors (Textor et al., 2016) (Supplementary Figure S2). Specifically, the analysis considered the following covariables: age (≤29, 30–34, and ≥35 years), sex (male, female), annual income (<30,000, 30,000 ~ 60,000, ≥60,000), education (middle school or below, high/ vocational school, college degree or above), passive smoking (never, occasionally, frequently), physical activity (low, moderate, vigorous), living children (yes or no), infertility treatment time (≤6, 7–12, 13–24, >24 months), cause of infertility (male, female, both, and unexplained), frequency of insomnia (never, occasionally or ≤3 per month, ≥4 per month), nocturnal wake frequency (never, occasionally, ≥1 per night), daytime napping (never, <1 and ≥1 h), social jetlag (<1 and ≥1 h), and nighttime sleep duration (<8 and ≥8 h).

### Statistical analysis

In the present analysis, continuous variables were expressed as mean ± standard deviation (SD) or median (P25, P75), whereas categorical variables were expressed as percentages. We conducted statistical analyses to examine the distribution of participants with various demographic characteristics based on their chronotypes (morning chronotype, intermediate chronotype, and evening chronotype). We utilized t-tests and analysis of variance (ANOVA) to analyze continuous variables, while Chi-square tests and Fisher’s exact test were employed for categorical variables. Given the small number of missing values, we address this issue by removing the corresponding rows. The study employed binary logistic regression models to investigate the association between chronotype and depression symptoms in individuals undergoing infertility treatment. We selected categorized depressive symptoms as the dependent variable and chronotype as the independent variable, with evening chronotype as the reference group in the binary logistic regression models. We used this group to calculate the odds ratios (ORs) and 95% confidence intervals (CIs) for the effect of chronotype on depression symptoms. The final model was adjusted for several potential confounding variables, including age, sex, annual income, education, passive smoking, physical activity, living children, infertility treatment time, cause of infertility, frequency of insomnia, nocturnal wake frequency, daytime napping, social jetlag, and nighttime sleep duration. Trend analyses were conducted by utilizing the median value of the various categories of chronotype scores as a continuous variable. An analysis using restrictive cubic splines with five knots was performed to describe the relationship between sleep and depression symptoms. We analyzed the combined effect of two variables and evaluated the association between chronotype and the likelihood of experiencing depression symptoms by categorizing the covariates mentioned earlier. A sensitivity analysis was conducted to assess the resilience of the findings by eliminating individuals who were engaged in shift work or night shifts. We employed the Actor-Partner Interdependence Model (APIM) approach to investigate the influence of one’s chronotype on one’s spouse’s depression symptoms, as suggested by Kenny et al. (2006). The APIM was implemented through multilevel modelling (MLM) with distinguishable dyads, treating gender as a distinguishing variable. PHQ-9 and rMEQ scores were included as continuous variables in the model for analysis, which adjusted for confounding variables consistent with the primary analysis. In the present study, the term “actor effect” refers to the impact of an individual’s chronotype on their depression symptoms, while the term “partner effect” refers to the impact of an individual’s chronotype on their spouse’s depression symptoms. Supplementary Figure S3 depicts the APIM framework for a husband-wife dyad. The statistical analyses mentioned above were conducted using R software version 4.1.0 (University of Auckland, Auckland, New Zealand) and SPSS version 23.0 (SPSS, Chicago, IL, United States). In this study, all statistical tests were double-tailed, and an a *p*-value less than 0.05 was considered statistically significant.

## Results

### General characteristics of participants across chronotype

Table 1 depicts the characteristics of the 1,022 (96.2%) participants included in the present study. Overall, participants included a total of 608 females (59.5%) and 514 males (40.5%), with 44.7% (*n* = 457) of individuals aged 30 to 34 years. According to the rMEQ scores, approximately 9.39% (95%CI: 9.82–10.33) of the participants were identified as having an evening chronotype, while 68.2% (95%CI: 14.75–14.97) had an intermediate chronotype and 29.06% (95%CI: 19.09–19.47) had a morning chronotype. Significant differences were found for age, passive smoking, education, annual income, frequency of insomnia, daytime napping, and living children compared to these three groups (all *p* < 0.05). Specifically, participants with an evening chronotype were found to be younger, free from passive smoking, with a higher level of education (either high school or vocational school), a higher annual income, more frequent insomnia, no daytime napping, lower levels of physical activity, longer sleep and social jetlag times, and shorter infertility treatment times. Furthermore, we noted that 527 participants exhibited mild or severe depression symptoms, accounting for 51.6% of the entire sample. There was a noticeable decrease in depression symptoms as the chronotype shifted from evening to intermediate and finally to morning. Supplementary Table S1 presents the characteristics of participants according to their depression phenotypes.

**Table 1:** The characteristics of participants according to the reduced Morningness-Eveningness Questionnaire (rMEQ) score status^†^.

Characteristic	Overall	Chronotypes^‡^			*p*-value
		Evening chronotype	Intermediate chronotype	Morning chronotype	
No. of participants	1,022	96(9.4)	697(68.2)	229(22.4)	
Age (%)					0.010
≤29	377 (36.9)	41 (42.7)	268 (38.5)	68 (29.7)	
30–34	457 (44.7)	46 (47.9)	305 (43.8)	106 (46.3)	
≥35	188 (18.4)	9 (9.4)	124 (17.8)	55 (24.0)	
Sex (Female, %)	608 (59.5)	56 (58.3)	428 (61.4)	124 (54.1)	0.148
Education (%)					0.011
Middle school or below	302 (29.5)	24 (25.0)	190 (27.3)	88 (38.4)	
High/vocational school	382 (37.4)	43 (44.8)	263 (37.7)	76 (33.2)	
College degree or above	338 (33.1)	29 (30.2)	244 (35.0)	65 (28.4)	
Annual incomes (%)					0.043
<30, 000	388 (38.0)	30 (31.2)	255 (36.6)	103 (45.0)	
30, 000–60, 000	242 (23.7)	23 (24.0)	178 (25.5)	41 (17.9)	
60, 000	392 (38.4)	43 (44.8)	264 (37.9)	85 (37.1)	
Passive smoking (%)					<0.001
Never	326 (31.9)	49 (51.0)	220 (31.6)	57 (24.9)	
Occasionally	591 (57.8)	45 (46.9)	419 (60.1)	127 (55.5)	
Frequently	105 (10.3)	2 (2.1)	58 (8.3)	45 (19.7)	
Physical activity (%)					0.467
Low	342 (33.5)	34 (35.4)	223 (32.0)	85 (37.1)	
Moderate	341 (33.4)	31 (32.3)	244 (35.0)	66 (28.8)	
Vigorous	339 (33.2)	31 (32.3)	230 (33.0)	78 (34.1)	
Cause of infertility (%)					0.923
Male factor	179 (17.5)	14 (14.6)	126 (18.1)	39 (17.0)	
Female factor	365 (35.7)	39 (40.6)	242 (34.7)	84 (36.7)	
Both	249 (24.4)	21 (21.9)	174 (25.0)	54 (23.6)	
Unexplained	229 (22.4)	22 (22.9)	155 (22.2)	52 (22.7)	
Living children (yes, %)	162 (15.9)	9 (9.4)	105 (15.1)	48 (21.0)	0.020
Infertility treatment time					0.335
≤6 month	342 (33.5)	40 (41.7)	223 (32.0)	79 (34.5)	
7–12 month	217 (21.2)	17 (17.7)	152 (21.8)	48 (21.0)	
13–24 month	310 (30.3)	26 (27.1)	223 (32.0)	61 (26.6)	
>24 month	153 (15.0)	13 (13.5)	99 (14.2)	41 (17.9)	
Frequency of insomnia (%)					<0.001
Never	236 (23.1)	14 (14.6)	156 (22.4)	66 (28.8)	
Occasionally or ≤3 per month	687 (67.2)	60 (62.5)	476 (68.3)	151 (65.9)	
≥4 per month	99 (9.7)	22 (22.9)	65 (9.3)	12 (5.2)	
Nocturnal wake frequency (%)					0.087
Never	158 (15.5)	23 (24.0)	106 (15.2)	29 (12.7)	
Occasionally	471 (46.1)	42 (43.8)	327 (46.9)	102 (44.5)	
≥1 per night	393 (38.5)	31 (32.3)	264 (37.9)	98 (42.8)	
Daytime napping (%)					0.004
Never	295 (28.9)	41 (42.7)	199 (28.6)	55 (24.0)	
<1 h	525 (51.4)	33 (34.4)	366 (52.5)	126 (55.0)	
≥1 h	202 (19.8)	22 (22.9)	132 (18.9)	48 (21.0)	
Social jetlag (≥1 h, %)	211 (20.6)	29 (30.2)	147 (21.1)	35 (15.3)	0.009
Nighttime sleep duration (≥8 h, %)	680 (66.5)	71 (74.0)	454 (65.1)	155 (67.7)	0.210
Depression symptoms (yes, %)	527 (51.6)	68 (70.8)	364 (52.2)	95 (41.5)	<0.001

^†^Values were presented as mean ± SD or percentages; ^‡^Evening Chronotype: 4–11 score; Intermediate Chronotype: 12–17 score; Morning Chronotype: 18–25 score.

### Binary logistic regression models

We assessed the association of chronotype with the risk of depressive status during infertility treatment, as depicted in Table 2. Compared to participants in the evening chronotype group, intermediate chronotype (OR = 0.45, 95% *CI*: 0.28–0.72, *P*_trend_ <0.001) and morning chronotype (OR = 0.29, 95% *CI*: 0.18–0.49, *P*_trend_ <0.001) participants were significantly associated with lower risks of depression symptoms. Similarly, after adjusting for confounders, intermediate chronotype (OR = 0.47, 95% *CI*: 0.28–0.77, *P*_trend_ <0.001) and morning chronotypes (OR = 0.32, 95% *CI*: 0.18–0.57, *P*_trend_ <0.001) were still associated with lower risks of depressive status, although this risk reduction was attenuated. The adjusted ORs (95% CIs) were 0.69 (0.59–0.79, *p* < 0.001) for depression symptoms for each standard deviation increase in rMEQ scores.

**Table 2:** Odds ratios and 95% confidence intervals of chronotype and depression symptoms in infertility populations.

Depression symptoms	Chronotypes^†^			Per 1-SD	*P*_trend_ ^d^
	Evening chronotype	Intermediate chronotype	Morning chronotype		
No. of cases/participants	68/96	364/697	95/229		
Model 1^a^	Reference	0.45 (0.28–0.72)^⁎⁎^	0.29 (0.18–0.49)^⁎⁎⁎^	0.68 (0.60–0.78)^⁎⁎⁎^	<0.001
Model 2^b^	Reference	0.45 (0.28–0.73)^⁎⁎^	0.31 (0.18–0.53)^⁎⁎⁎^	0.68 (0.59–0.78)^⁎⁎⁎^	<0.001
Model 3^c^	Reference	0.47 (0.28–0.77)^⁎⁎^	0.32 (0.18–0.57)^⁎⁎⁎^	0.69 (0.59–0.79)^⁎⁎⁎^	<0.001

^†^Evening chronotype: 4–11 score; Intermediate chronotype: 12–17 score; Morning chronotype: 18–25 score. ^*^p < 0.05, ^**^p < 0.01, ^***^p < 0.001.

a Model 1: it did not adjust for the covariates.

b Model 2: it additionally included age, sex, education, annual incomes, passive smoking, physical activity, cause of infertility, living children, and infertility treatment time.

c Model 3: it additionally included frequency of insomnia, nocturnal wake frequency, daytime napping, social jetlag, and nighttime sleep duration.

d The trend test was performed by assigning medians into three groups and using them as a continuous variable in the models.

### Test for nonlinear association between rMEQ scores and depression symptoms

**Figure 1:**
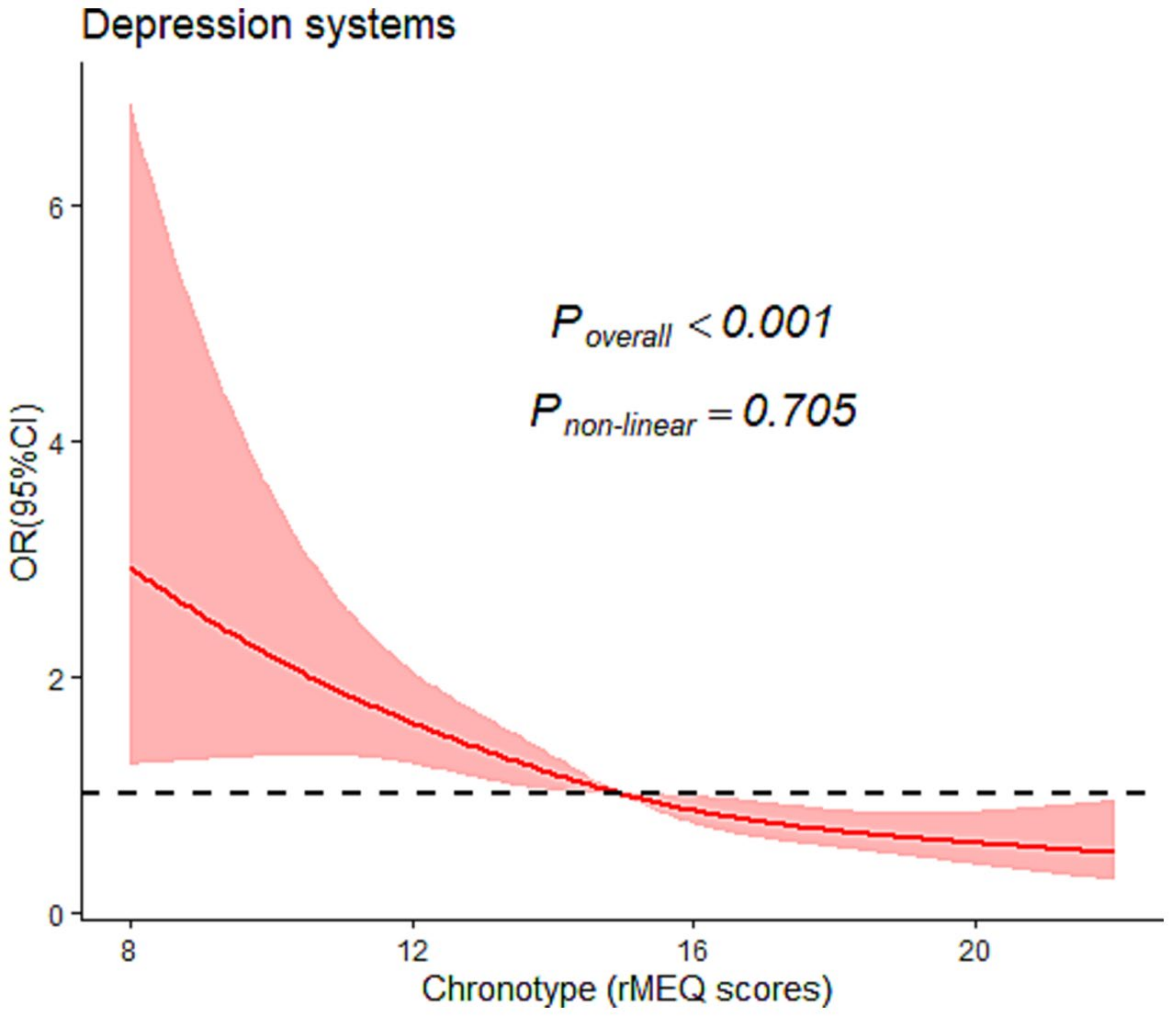
Graphical representation of the association between chronotype and the severity of their depression symptoms using restricted cubic splines. OR, Odds ratio; CI, confidence interval. Covariates adjusted in the models were the same as those of Model 3 in Table 2 (see Table 2 footnote).

Furthermore, we examined the non-linear dose–response relationship between chronotype (rMEQ score) and risk of depression symptoms by restrictive cubic splines, as shown in Figure 1. After adjusting various factors, such as age, sex, annual income, education, passive smoking, physical activity, living children, infertility treatment time, cause of infertility, frequency of insomnia, nocturnal wake frequency, daytime napping, social jetlag, and nighttime sleep duration, we found that the probability of depression symptoms seemed to decrease as rMEQ scores (*P*_overall_ <0.001) increased; however, no nonlinear trend was observed (*P*_non-linear_ = 0.526).

### Stratified and sensitivity analyses

**Figure 2:**
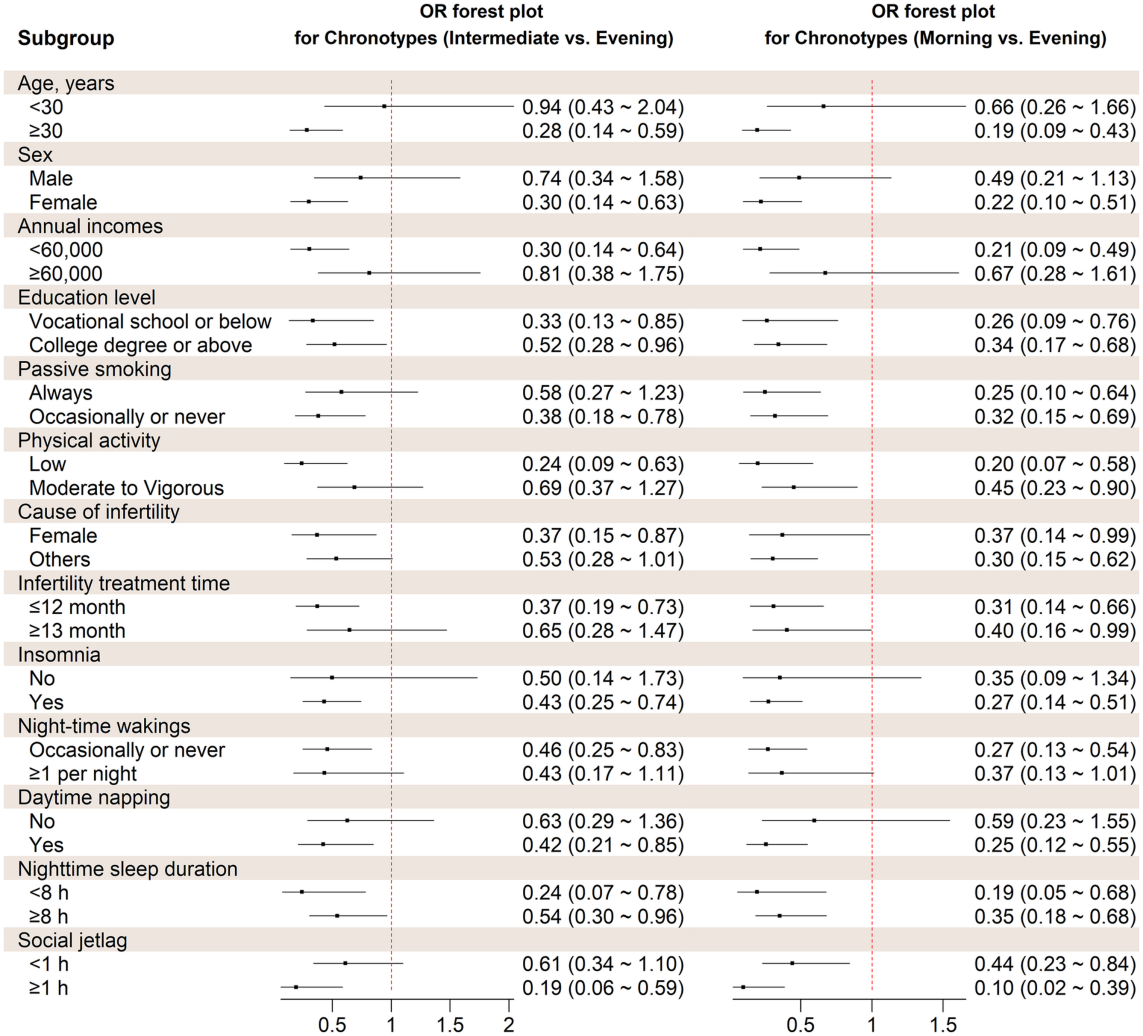
Stratified analysis on the association between chronotype and probability of depression symptoms. OR, Odds ratio. Covariates adjusted in the models were the same as those in Model 3 in Table 2 (see Table 2 footnote).

The stratified analysis revealed similar association between chronotype and the likelihood of experiencing depression symptoms across various factors, including age, sex, annual income, education, passive smoking, physical activity, living children, infertility treatment time, cause of infertility, frequency of insomnia, nocturnal wake frequency, daytime napping, social jetlag, and nighttime sleep duration (Figure 2). Based on interaction studies, it shows a slight interaction between age and chronotype (*P*_for interaction_ = 0.045). However, none of the other factors showed any interaction with sleep chronotype (*P*_for interaction_ > 0.05).

Furthermore, we conducted sensitivity analyses to assess the reliability of the findings by eliminating participants who worked shifts or nights. The relationship between morning and intermediate chronotypes and the likelihood of depressive status remained consistent with the main analysis (OR = 0.51, 95% *CI*: 0.30–0.87, *P*_trend_ <0.001; OR = 0.32, 95% *CI*: 0.18–0.58, *P*_trend_ <0.001, respectively). Supplementary Table S2 depicts the analysis.

### APIM analysis

We assessed 397 couples who could not conceive among 1,022 individuals to determine their eligibility for inclusion in the APIM with distinct dyads. After controlling for confounding variables, the findings indicated that the wife’s chronotype had a substantial impact on her depression symptoms (*β* = −0.154, *p* < 0.01), but the husband’s chronotype did not demonstrate a noteworthy influence on his depression symptoms. Moreover, contrary to our expectations, our study did not observe a strong partner effect of female or male chronotype on the depression symptoms of spouses (Table 3).

**Table 3:** Actor and partner effects of chronotype on depression symptoms in infertile couples (*n* = 397 couples).

Actor and partner effects	β (95% CIs)	
	Model 1^a^	Model 2^b^
Actor effect		
Wives’ chronotype → Wives’ depression	−0.197 (−0.296, −0.097)^⁎⁎⁎^	−0.154 (−0.254, −0.055)^⁎⁎^
Husbands’ chronotype patterns → Husbands’ depression	−0.090 (−0.177, −0.002)^⁎^	−0.070 (−0.154, 0.015)
Partner effect		
Wives’ chronotype → Husbands’ depression	−0.028 (−0.129, 0.072)	−0.049 (−0.145, 0.047)
Husbands’ chronotype → Wives’ depression	0.056 (−0.030, 0.143)	0.041 (−0.043, 0.125)

β, Standardized Coefficient; CIs, confidence intervals. ^⁎^*p* < 0.05, ^⁎⁎^*p* < 0.01, ^⁎⁎⁎^*p* < 0.001.

a Model 1: It did not adjust for the covariates.

b Model 2: Additionally it included age, sex, education, annual incomes, passive smoking, physical activity, cause of infertility, living children, infertility treatment time, frequency of insomnia, nocturnal wake frequency, daytime napping, social jetlag, and nighttime sleep duration.

## Discussion

### Primary findings

This study examined 1,022 Chinese infertility patients to investigate the relationship between chronotype and depression symptoms during assisted reproductive treatment. The findings revealed that individuals with morning and intermediate chronotypes were less likely to experience depression symptoms compared to those with an evening chronotype. This association was determined through a comparative analysis of chronotype and PHQ-9 scores. Furthermore, the protective effect of morning and intermediate chronotypes was more pronounced in individuals aged 30 years or older and those with a nighttime sleep duration of less than 8 h or social jetlag of 1 h or more. This is the first study to examine the connection between chronotype and depressed mood in individuals undergoing assisted reproductive technology treatment for infertility. Nevertheless, it is important to exercise caution when interpreting the findings, as the chronotype was determined using self-reported rMEQ scores, which have the potential for misclassification.

### Interpretation

There is growing evidence that evening chronotype is significantly associated with negative effects, which is shown not only in the general population but also in specific groups such as the elderly, pregnant women, children, and adolescents, aligning with the present findings. Similar findings were observed in a Mendelian randomization study, where earlier diurnal preference was shown to be associated with a 23% lower risk of major depressive disorder (Daghlas et al., 2021). Furthermore, a study conducted on animals investigating the effects of cortisol-induced depression-like behavior indicated that circadian rhythms may be a causal factor or a predictor of depression-like episodes (Spulber et al., 2015). Nevertheless, a recently published longitudinal study found no association between chronotype and the duration of depression or anxiety disorders (Druiven et al., 2019). The disparities above may arise from variations in the study participants, the extent to which findings from animal experiments can be directly extrapolated to humans, and the accuracy of chronotype in predicting the progression of depressive illness. Additional clinical trials or cohort studies are necessary to explore these health impacts. Furthermore, Chronotype is not only linked to the diagnosis of a depressive disease but also to the extent or intensity of the disorder (Druiven et al., 2019). Remarkably, a study conducted over 7 years revealed that alterations in chronotype were solely linked to the intensity of depression symptoms while not correlated with anxious symptoms (Druiven et al., 2020). Furthermore, the distinct influence of chronotype has also been reported. A study found that excessive drowsiness throughout the day and accumulated sleep debt had a role in the impact of evening chronotype preferences among college students in their early adulthood, enhancing the risk of anxiety and depression. However, these effects were not observed in the wider population of young adults (Dickinson et al., 2018). Furthermore, research has verified that individuals with an evening chronotype are more prone to experiencing depression compared to individuals with other chronotypes. This association remains significant regardless of perceived stress, inadequate sleep quality, and insufficient sleep duration (Merikanto et al., 2015; Tonon et al., 2020). It has to be mentioned that unlike unidimensional measures of morningness-eveningness, the Morningness-Eveningness Stability Scale improved (MESSi) can assess multi-dimensions of chronotype, including morning affect, eveningness, and amplitude of diurnal variation (distinctness), respectively (Vagos et al., 2019). Similar to the present study, one study suggested that morning affect was significantly inversely correlated with childhood depression (İpar, 2023). Another study also found that negative emotionality (including depression, anxiety, and stress) was positively correlated with morning affect and inversely correlated with eveningness and distinctness. However, after controlling for morning affect, a correlation between negative emotionality and eveningness was not observed (Carciofo, 2020). To a certain extent, both morning affect and a stronger amplitude of diurnal variation may be more strongly associated with negative emotionality than eveningness preference, possibly due to distinctiveness as a component of chronotype, which has an important impact on susceptibility to depression. MESSi proposes a multidimensional measurement framework, which will undoubtedly provide greater nuance and support for assessing circadian preferences in the future. Although chronotype can be assessed by different biological and objective indicators, self-report questionnaires are still widely used. Accurately measuring this individual chronotype variation is an important aspect, so this remains for future research. Undoubtedly, the results of these studies emphasize the significance of investigating the influence of chronotype on depression. Depression negatively impacts pregnancy outcomes during IVF-ET treatment (Cui et al., 2020). Infertility patients can enhance the results of infertility therapy by adopting certain lifestyle modifications, such as keeping a morning or intermediate chronotype and avoiding an evening chronotype. Thus, they can improve their depression state while undergoing ART treatment. Furthermore, from a clinical standpoint, the current study has the potential to provide valuable insights for creating tailored therapies for healthcare providers. Interventions can effectively address psychological issues like depression, while demographic and gynecological characteristics are not amenable to such interventions. Hence, chronotype serves as a valuable modifiable factor in infertility treatment that can be assessed before commencing infertility treatment. Enhancing the reproductive outcomes of infertility patients can be achieved by effectively regulating their chronotype, which in turn helps them cope with infertility and associated medical procedures, hence alleviating depression symptoms.

The present results confirmed and extended previous epidemiological studies showing the sex-specific effects of chronotype on depression status. A study conducted in Korea among 5,550 non-shift working people revealed that having a late chronotype was linked to a 2.9-fold higher likelihood of experiencing depression in women but not in men (Kim et al., 2020). The present findings coincide closely with previous studies and extend these observations further by including a larger population experiencing infertility. Specifically, we found through APIM that one’s depression symptoms may not be influenced by the chronotype of the partner, which is similar to the results of our stratified analyses. Our research revealed a noteworthy correlation between age and chronotype in the likelihood of experiencing depression symptoms. Specifically, we found that individuals aged 30 years or older who identified as morning or intermediate chronotypes noticed health benefits to depression symptoms. Genetic and age-related factors determine the chronotype, which tends to shift towards earlier times as a person ages (Taillard et al., 2021). Typically, adolescents and young adults tend to have an evening chronotype (Keller et al., 2016). According to a study conducted on a mostly rural population, there was a significant association between mild to severe depression and a tendency to have a later sleep–wake cycle and experience a greater difference in sleep patterns between workdays and free days (social jetlag of more than 2 h). This association was particularly strong among individuals aged 31–40 years (Levandovski et al., 2011), consistent with the findings of the present study. Furthermore, we have noticed that a lack of sleep and experiencing significant differences in sleep patterns due to social obligations can also contribute to depression symptoms. Specifically, individuals who have a preference for waking up early in the morning or have an intermediate preference for waking up have greater improvements in their depression symptoms if they also have a social jetlag of 1 h or more and sleep less than 8 h. Numerous epidemiological studies have investigated the correlation between sleep deprivation and depression. These studies have consistently demonstrated that sleep deprivation is a significant risk factor for the onset of depression. Furthermore, it is believed that a sleep disturbance may contribute to this association. Furthermore, a recent study has demonstrated a substantial correlation between social jetlag and depression, which is not influenced by sleep debt (Okajima et al., 2021). While it aligns largely with our conjecture, additional clinical trials or cohort studies are necessary to substantiate these health impacts in the future.

While the exact mechanisms between chronotype and depression symptoms remain unclear, there are potential explanations. Circadian rhythms were suggested to play an essential role in this association. One line of thinking suggests that circadian clock polymorphisms associated with both chronotype and depression symptoms (Molina-Montes et al., 2022; Zou et al., 2022; Dang et al., 2023; Li et al., 2023). Mutations in specific clock genes may cause the sleep–wake cycle to phase shift, disrupting activity with the neurotransmitter system, leading to a greater likelihood of nocturnal preference and subsequent depressive symptoms (Au and Reece, 2017). However, the molecular and physiological mechanisms are unclear, particularly how clock gene polymorphisms contribute to depression. Another potential explanatory mechanism is circadian rhythm disorder or social jet lag, which refers to changes in the sleep–wake cycle caused by exogenous factors, followed by sleep disorders. This promotes the onset and exacerbates the severity of depression (Pandi-Perumal et al., 2020). Furthermore, psychological mechanisms for the association between chronotype and depression symptoms may include cognitive emotion regulation (Van den Berg et al., 2018). It has been suggested that individuals with evening chronotype may be more prone to specific thought patterns at night, and thus have higher psychological vulnerability (e.g., cognitive reactivity), or carry personality traits that put them at risk (e.g., neuroticism), making them more susceptible to depression (Wittmann et al., 2006; Antypa et al., 2017). Related neurobiological explanations focused on structural and functional brain abnormalities. First, previous studies have extensively shown that the responsiveness of the amygdala plays a vital role in determining emotional consequences (Schiel et al., 2022). Individuals with a later chronotype showed increased amygdala sensitivity to negative emotional stimuli. Similar results have been observed in individuals with depression and high-risk populations, such as those with high levels of anxiety and a family history of depression. Second, besides the level of amygdala activity associated with emotional processing, there are variations in the functional connection between the amygdala and dorsal anterior cingulate cortex. Horne and colleagues found a significant reduction in the functional connection between the amygdala and dorsal anterior cingulate cortex in individuals with a later chronotype (Horne and Norbury, 2018). The evening chronotype is associated with a higher emotional reaction to negative stimuli due to the inhibition of the dorsal anterior cingulate regulation of the amygdala, impacting the modulation of emotions. For individuals undergoing assisted reproductive technology treatments, who themselves suffer from a long history of many stressful fertility events and cognitive-emotional regulation difficulties, there is a higher likelihood of exacerbating the severity of difficulty falling asleep, insomnia, and poor sleep quality. In addition, individuals with a nocturnal preference are more likely to worry about clinical appointments and about their own sleep habits deviating from those required for healthy reproduction, thereby disrupting their sleep–wake patterns and biological clocks, which in turn may increase the risk of depression. Nevertheless, the underlying causes of depression symptoms can originate from multiple factors in the context of infertility treatment. Further research is required to confirm this connection and find efficient methods to enhance mental well-being among individuals with infertility.

### Strengths and limitations

The current study has several strengths in terms of its techniques and design. One of these strengths is the selection of infertile couples with well-documented psychological traits as research subjects. This selection helps to enhance the trustworthiness of the research results to a certain extent. About exposure evaluation, we methodically gathered information about sleep characteristics using the 22-item Sleep Factor Questionnaire (SFQ) (Yang et al., 2017). Additionally, we considered other sleep-related variables that could potentially affect the occurrence of depression in our research. We were the first group to investigate the connections between chronotype and depression symptoms in infertile couples. However, we did not see any impacts of interdependence between partners.

There are certain limitations to the present study. First, our strategy involved cross-sectional research instead of longitudinal studies or randomized clinical trials. Cross-sectional studies can describe connections but are restricted in their ability to establish causal inference. Nevertheless, this study can still offer insights into the risk factors associated with depression symptoms and potentially serve as the scientific foundation for future investigations. Second, the participants’ chronotype and depression symptoms were assessed through self-reporting throughout ovulation induction treatment using established scales that demonstrated high reliability and validity. However, it is important to acknowledge that this method may introduce bias and may weaken the strength of our findings in certain aspects. Third, this study is limited to a single center and focuses on couples undergoing infertility treatment, which could introduce selection bias. The results may not be directly applicable to other populations. Additional multicenter studies could be undertaken to substantiate our findings further. Fourth, the data of the present study were collected online, which may have affected the reliability of participants’ answers. However, online data collection is being used increasingly, especially in sleep medicine. Despite some limitations, large amounts of data were accessed quickly and at a low cost in the context of COVID-19 (Uygur et al., 2022). Also, the present study underwent rigorous quality control to ensure that online data collection was as valid and reliable as traditional data collection methods, including but not limited to trained data collectors to assist field participants with accurate recording, standardization of the data collection process, and pre-survey. Fifth, the sleep characteristics reported in this study were derived from the preceding 6 months, perhaps failing to accurately represent long-term sleep patterns. Furthermore, this study does not provide objective indicators to elucidate the molecular mechanism behind the correlation between chronotype and depression symptoms in infertile couples. Therefore, future studies should aim to provide further validation.

## Conclusion

This study found a significant association between chronotype and depression symptoms in infertility patients without the partner effect in couples. Chronotype is a crucial issue to consider in the field of reproduction. Further large-scale investigations are required to clarify the impact of chronotype on reproductive processes and treatment outcomes through the modification of depression symptoms.

## Data Availability

The raw data supporting the conclusions of this article will be made available by the authors, without undue reservation.

## References

[ref1] Adan A. Almirall H. (1992). The influence of age, work schedule and personality on morningness dimension. Int. J. Psychophysiol. 12, 95–99. doi: 10.1016/0167-8760(92)90001-r, PMID: 1592674

[ref2] Agarwal A. Majzoub A. Parekh N. Henkel R. (2020). A schematic overview of the current status of male infertility practice. World J Mens Health 38, 308–322. doi: 10.5534/wjmh.190068, PMID: 31385475 PMC7308239

[ref3] Aimagambetova G. Issanov A. Terzic S. Bapayeva G. Ukybassova T. Baikoshkarova S.. (2020). The effect of psychological distress on IVF outcomes: reality or speculations? PLoS One 15:e0242024. doi: 10.1371/journal.pone.0242024, PMID: 33315878 PMC7735622

[ref4] Antypa N. Verkuil B. Molendijk M. Schoevers R. Penninx B. Van Der Does W. (2017). Associations between chronotypes and psychological vulnerability factors of depression. Chronobiol. Int. 34, 1125–1135. doi: 10.1080/07420528.2017.1345932 28759270

[ref5] Antypa N. Vogelzangs N. Meesters Y. Schoevers R. Penninx B. W. (2016). Chronotype associations with depression and anxiety disorders in a large cohort study. Depress. Anxiety 33, 75–83. doi: 10.1002/da.22422, PMID: 26367018

[ref6] Au J. Reece J. (2017). The relationship between chronotype and depressive symptoms: a meta-analysis. J. Affect. Disord. 218, 93–104. doi: 10.1016/j.jad.2017.04.021, PMID: 28463712

[ref7] Caci H. Deschaux O. Adan A. Natale V. (2009). Comparing three morningness scales: age and gender effects, structure and cut-off criteria. Sleep Med. 10, 240–245. doi: 10.1016/j.sleep.2008.01.007, PMID: 18387342

[ref8] Caetano G. Bozinovic I. Dupont C. Léger D. Lévy R. Sermondade N. (2021). Impact of sleep on female and male reproductive functions: a systematic review. Fertil. Steril. 115, 715–731. doi: 10.1016/j.fertnstert.2020.08.1429, PMID: 33054981

[ref9] Carciofo R. (2020). Morning affect, eveningness, and amplitude distinctness: associations with negative emotionality, including the mediating roles of sleep quality, personality, and metacognitive beliefs. Chronobiol. Int. 37, 1565–1579. doi: 10.1080/07420528.2020.1798978, PMID: 32787687

[ref10] Carciofo R. Du F. Song N. Qi Y. Zhang K. (2012). Age-related chronotype differences in Chinese, and reliability assessment of a reduced version of the Chinese Morningness-Eveningness questionnaire. Sleep Biol. Rhythms 10, 310–318. doi: 10.1111/j.1479-8425.2012.00577.x

[ref11] Cesta C. E. Viktorin A. Olsson H. Johansson V. Sjölander A. Bergh C.. (2016). Depression, anxiety, and antidepressant treatment in women: association with in vitro fertilization outcome. Fertil. Steril. 105, 1594–1602.e3. doi: 10.1016/j.fertnstert.2016.01.036, PMID: 26920258

[ref12] Cocchiaro T. Meneghini C. Dal Lago A. Fabiani C. Amodei M. Miriello D.. (2020). Assessment of sexual and emotional distress in infertile couple: validation of a new specific psychometric tool. J. Endocrinol. Investig. 43, 1729–1737. doi: 10.1007/s40618-020-01263-z, PMID: 32333331

[ref13] Codoner-Franch P. Gombert M. Martinez-Raga J. Cenit M. C. (2023). Circadian disruption and mental health: the Chronotherapeutic potential of microbiome-based and dietary strategies. Int. J. Mol. Sci. 24:579. doi: 10.3390/ijms24087579, PMID: 37108739 PMC10146651

[ref14] Comas M. Solis Flores A. Lovato N. Miller C. B. Bartlett D. J. Grunstein R. R.. (2023). The relationship between anxiety, subjective and objective sleep, Chronotype and circadian rhythms with depressive symptoms in insomnia disorder. Brain Sci. 13:613. doi: 10.3390/brainsci13040613, PMID: 37190578 PMC10136589

[ref15] Coward R. M. Stetter C. Kunselman A. Trussell J. C. Lindgren M. C. Alvero R. R.. (2019). Fertility related quality of life, gonadal function and erectile dysfunction in male Partners of Couples with unexplained infertility. J. Urol. 202, 379–384. doi: 10.1097/ju.0000000000000205, PMID: 30835629 PMC6686175

[ref16] Cui Y. Yu H. Meng F. Liu J. Yang F. (2020). Prospective study of pregnancy outcome between perceived stress and stress-related hormones. J. Obstet. Gynaecol. Res. 46, 1355–1363. doi: 10.1111/jog.14278, PMID: 32500644

[ref17] Daghlas I. Lane J. M. Saxena R. Vetter C. (2021). Genetically Proxied diurnal preference, sleep timing, and risk of major depressive disorder. JAMA Psychiatry 78, 903–910. doi: 10.1001/jamapsychiatry.2021.0959, PMID: 34037671 PMC8156187

[ref18] Dang T. Russel W. A. Saad T. Dhawka L. Ay A. Ingram K. K. (2023). Risk for seasonal affective disorder (SAD) linked to circadian clock gene variants. Biology 12:532. doi: 10.3390/biology12121532, PMID: 38132358 PMC10741218

[ref19] Danielsson K. Sakarya A. Jansson-Fröjmark M. (2019). The reduced Morningness-Eveningness questionnaire: psychometric properties and related factors in a young Swedish population. Chronobiol. Int. 36, 530–540. doi: 10.1080/07420528.2018.1564322, PMID: 30614272

[ref20] Dickinson D. L. Wolkow A. P. Rajaratnam S. M. W. Drummond S. P. A. (2018). Personal sleep debt and daytime sleepiness mediate the relationship between sleep and mental health outcomes in young adults. Depress. Anxiety 35, 775–783. doi: 10.1002/da.22769, PMID: 29790238

[ref21] Druiven S. J. M. Hovenkamp-Hermelink J. H. M. Knapen S. E. Kamphuis J. Haarman B. C. M. Penninx B.. (2020). Stability of chronotype over a 7-year follow-up period and its association with severity of depressive and anxiety symptoms. Depress. Anxiety 37, 466–474. doi: 10.1002/da.22995, PMID: 32065480 PMC7318352

[ref22] Druiven S. J. M. Knapen S. E. Penninx B. Antypa N. Schoevers R. A. Riese H.. (2019). Can chronotype function as predictor of a persistent course of depressive and anxiety disorder? J. Affect. Disord. 242, 159–164. doi: 10.1016/j.jad.2018.08.064, PMID: 30179789

[ref23] Evans-Hoeker E. A. Eisenberg E. Diamond M. P. Legro R. S. Alvero R. Coutifaris C.. (2018). Major depression, antidepressant use, and male and female fertility. Fertil. Steril. 109, 879–887. doi: 10.1016/j.fertnstert.2018.01.029, PMID: 29778387 PMC5973807

[ref24] Ferriani L. O. Silva D. A. Molina M. Mill J. G. Brunoni A. R. Da Fonseca M. J. M.. (2023). Depression is a risk factor for metabolic syndrome: results from the ELSA-Brasil cohort study. J. Psychiatr. Res. 158, 56–62. doi: 10.1016/j.jpsychires.2022.12.017 36571912

[ref25] Friedrich M. J. (2017). Depression is the leading cause of disability around the world. JAMA 317:1517. doi: 10.1001/jama.2017.3826, PMID: 28418490

[ref26] Gica S. Demirkol M. K. Yildirim A. Temiz Dogan N. Resim S. (2023). Evening type negatively affects semen quality by deteriorating sperm morphology: results from an infertility clinic. Eur. J. Obstet. Gynecol. Reprod. Biol. 291, 190–195. doi: 10.1016/j.ejogrb.2023.10.019, PMID: 38353088

[ref27] Giesinger J. M. Kieffer J. M. Fayers P. M. Groenvold M. Petersen M. A. Scott N. W.. (2016). Replication and validation of higher order models demonstrated that a summary score for the EORTC QLQ-C30 is robust. J. Clin. Epidemiol. 69, 79–88. doi: 10.1016/j.jclinepi.2015.08.007, PMID: 26327487

[ref28] Horne C. M. Norbury R. (2018). Late chronotype is associated with enhanced amygdala reactivity and reduced fronto-limbic functional connectivity to fearful versus happy facial expressions. NeuroImage 171, 355–363. doi: 10.1016/j.neuroimage.2018.01.025, PMID: 29339309

[ref29] Horne J. A. Ostberg O. (1976). A self-assessment questionnaire to determine Morningness-Eveningness in human circadian rhythms. Int. J. Chronobiol. 4, 97–110. 1027738

[ref30] Inhorn M. C. Patrizio P. (2015). Infertility around the globe: new thinking on gender, reproductive technologies and global movements in the 21st century. Hum. Reprod. Update 21, 411–426. doi: 10.1093/humupd/dmv016, PMID: 25801630

[ref31] İpar N. (2023). The effect of circadian preference and sleep disturbances on depression in children 6 to 12 years of age. Chronobiol. Int. 40, 1375–1386. doi: 10.1080/07420528.2023.2262577, PMID: 37781873

[ref32] Kamboj N. Saraswathy K. N. Prasad S. Babu N. Puri M. Sharma A.. (2023). Women infertility and common mental disorders: a cross-sectional study from North India. PLoS One 18:e0280054. doi: 10.1371/journal.pone.0280054, PMID: 36603005 PMC9815660

[ref33] Keller L. K. Zöschg S. Grünewald B. Roenneberg T. Schulte-Körne G. (2016). Chronotype and depression in adolescents – a review. Z. Kinder Jugendpsychiatr. Psychother. 44, 113–126. doi: 10.1024/1422-4917/a000406, PMID: 27008901

[ref34] Kenny D. A. Kashy D. A. Cook W. L. (2006). Dyadic data analysis. New York: Guilford Press.

[ref35] Kim K. M. Han S. M. Heo K. Kim W. J. Chu M. K. (2020). Sex differences in the association between chronotype and risk of depression. Sci. Rep. 10:18512. doi: 10.1038/s41598-020-75724-z, PMID: 33116223 PMC7595163

[ref36] Kroenke K. (2021). PHQ-9: global uptake of a depression scale. World Psychiatry 20, 135–136. doi: 10.1002/wps.20821, PMID: 33432739 PMC7801833

[ref37] Lang J. Zhang B. Meng Y. Du Y. Cui L. Li W. (2019). First trimester depression and/or anxiety disorders increase the risk of low birthweight in IVF offspring: a prospective cohort study. Reprod. Biomed. Online 39, 947–954. doi: 10.1016/j.rbmo.2019.09.002, PMID: 31734092

[ref38] Levandovski R. Dantas G. Fernandes L. C. Caumo W. Torres I. Roenneberg T.. (2011). Depression scores associate with chronotype and social jetlag in a rural population. Chronobiol. Int. 28, 771–778. doi: 10.3109/07420528.2011.602445, PMID: 21895489

[ref39] Li J. Huang Y. Xu S. Wang Y. (2024). Sleep disturbances and female infertility: a systematic review. BMC Womens Health 24:643. doi: 10.1186/s12905-024-03508-y, PMID: 39707272 PMC11660991

[ref40] Li Y. Ma J. Yao K. Su W. Tan B. Wu X.. (2020). Circadian rhythms and obesity: timekeeping governs lipid metabolism. J. Pineal Res. 69:e12682. doi: 10.1111/jpi.12682, PMID: 32656907

[ref41] Li Y. Miao P. Li F. Huang J. Fan L. Chen Q.. (2023). An association study of clock genes with major depressive disorder. J. Affect. Disord. 341, 147–153. doi: 10.1016/j.jad.2023.08.113, PMID: 37633529

[ref42] Liu Z. Zheng Y. Wang B. Li J. Qin L. Li X.. (2023). The impact of sleep on in vitro fertilization embryo transfer outcomes: a prospective study. Fertil. Steril. 119, 47–55. doi: 10.1016/j.fertnstert.2022.10.015, PMID: 36435629

[ref43] Lotti S. Dinu M. Colombini B. Amedei A. Sofi F. (2023). Circadian rhythms, gut microbiota, and diet: possible implications for health. Nutr. Metab. Cardiovasc. Dis. 33, 1490–1500. doi: 10.1016/j.numecd.2023.05.009, PMID: 37246076

[ref44] Löwe B. Kroenke K. Herzog W. Gräfe K. (2004). Measuring depression outcome with a brief self-report instrument: sensitivity to change of the patient health questionnaire (PHQ-9). J. Affect. Disord. 81, 61–66. doi: 10.1016/s0165-0327(03)00198-8, PMID: 15183601

[ref45] Lu S. Ma Z. Zhou W. Zeng H. Ma J. Deng H.. (2024). Association of sleep traits with male fertility: a two-sample Mendelian randomization study. Front. Genet. 15:1353438. doi: 10.3389/fgene.2024.1353438, PMID: 38456015 PMC10917924

[ref46] Malin S. K. Remchak M. E. Heiston E. M. Battillo D. J. Gow A. J. Shah A. M.. (2024). Intermediate versus morning chronotype has lower vascular insulin sensitivity in adults with obesity. Diabetes Obes. Metab. 26:456. doi: 10.1111/dom.15456, PMID: 38246697 PMC11001524

[ref47] Mentzelou M. Papadopoulou S. K. Papandreou D. Spanoudaki M. Dakanalis A. Vasios G. K.. (2023). Evaluating the relationship between circadian rhythms and sleep, metabolic and cardiovascular disorders: current clinical evidence in human studies. Meta 13:370. doi: 10.3390/metabo13030370, PMID: 36984810 PMC10057970

[ref48] Merikanto I. Kronholm E. Peltonen M. Laatikainen T. Vartiainen E. Partonen T. (2015). Circadian preference links to depression in general adult population. J. Affect. Disord. 188, 143–148. doi: 10.1016/j.jad.2015.08.061, PMID: 26363264

[ref49] Molina-Montes E. Rodríguez-Barranco M. Ching-López A. Artacho R. Huerta J. M. Amiano P.. (2022). Circadian clock gene variants and their link with chronotype, chrononutrition, sleeping patterns and obesity in the European prospective investigation into cancer and nutrition (EPIC) study. Clin. Nutr. 41, 1977–1990. doi: 10.1016/j.clnu.2022.07.027, PMID: 35961261

[ref50] Monteggia L. M. Kavalali E. T. (2012). Circadian rhythms: depression brought to light. Nature 491, 537–538. doi: 10.1038/nature11752, PMID: 23151474

[ref51] Okajima I. Komada Y. Ito W. Inoue Y. (2021). Sleep debt and social jetlag associated with sleepiness, mood, and work performance among Workers in Japan. Int. J. Environ. Res. Public Health 18:908. doi: 10.3390/ijerph18062908, PMID: 33809121 PMC8000941

[ref52] Ozcelik C. Varli B. Gokce A. Takmaz T. Cetin C. Ozcan P. (2023). Evaluation of chronotype and sleep quality in infertile population and comparison with fertile population: a cross-sectional study. J. Psychosom. Obstet. Gynaecol. 44:2148523. doi: 10.1080/0167482X.2022.2148523, PMID: 36480727

[ref53] Pandi-Perumal S. R. Monti J. M. Burman D. Karthikeyan R. Bahammam A. S. Spence D. W.. (2020). Clarifying the role of sleep in depression: a narrative review. Psychiatry Res. 291:113239. doi: 10.1016/j.psychres.2020.113239, PMID: 32593854

[ref54] Park H. Lee H. K. Lee K. (2018). Chronotype and suicide: the mediating effect of depressive symptoms. Psychiatry Res. 269, 316–320. doi: 10.1016/j.psychres.2018.08.046, PMID: 30172189

[ref55] Peng X. Fan R. Xie L. Shi X. Dong K. Zhang S.. (2022). A growing link between circadian rhythms, type 2 diabetes mellitus and Alzheimer's disease. Int. J. Mol. Sci. 23:504. doi: 10.3390/ijms23010504, PMID: 35008933 PMC8745289

[ref56] Purewal S. Chapman S. C. E. Van Den Akker O. B. A. (2018). Depression and state anxiety scores during assisted reproductive treatment are associated with outcome: a meta-analysis. Reprod. Biomed. Online 36, 646–657. doi: 10.1016/j.rbmo.2018.03.010, PMID: 29622404

[ref57] Ramzi N. H. Auvinen J. Veijola J. Miettunen J. Ala-Mursula L. Sebert S.. (2023). Depression mediates the relationship between alexithymia and obesity in the northern Finland birth cohort 1966 (NFBC1966). J. Affect. Disord. 331, 1–7. doi: 10.1016/j.jad.2023.03.026, PMID: 36933669

[ref58] Refisch A. Sen Z. D. Klassert T. E. Busch A. Besteher B. Danyeli L. V.. (2023). Microbiome and immuno-metabolic dysregulation in patients with major depressive disorder with atypical clinical presentation. Neuropharmacology 235:109568. doi: 10.1016/j.neuropharm.2023.109568, PMID: 37182790

[ref59] Roenneberg T. Kuehnle T. Juda M. Kantermann T. Allebrandt K. Gordijn M.. (2007). Epidemiology of the human circadian clock. Sleep Med. Rev. 11, 429–438. doi: 10.1016/j.smrv.2007.07.005, PMID: 17936039

[ref60] Schiel J. E. Tamm S. Holub F. Petri R. Dashti H. S. Domschke K.. (2022). Associations between sleep health and amygdala reactivity to negative facial expressions in the UK biobank cohort. Biol. Psychiatry 92, 693–700. doi: 10.1016/j.biopsych.2022.05.023, PMID: 35933167 PMC13375863

[ref61] Sejbaek C. S. Hageman I. Pinborg A. Hougaard C. O. Schmidt L. (2013). Incidence of depression and influence of depression on the number of treatment cycles and births in a national cohort of 42,880 women treated with ART. Hum. Reprod. 28, 1100–1109. doi: 10.1093/humrep/des442, PMID: 23300199

[ref62] Senesi P. Ferrulli A. Luzi L. Terruzzi I. (2022). Chrono-communication and cardiometabolic health: the intrinsic relationship and therapeutic nutritional promises. Front. Endocrinol. 13:975509. doi: 10.3389/fendo.2022.975509, PMID: 36176473 PMC9513421

[ref63] Shah R. M. Doshi S. Shah S. Patel S. Li A. Diamond J. A. (2023). Impacts of anxiety and depression on clinical hypertension in low-income US adults. High Blood Press Cardiovasc. Prev. 30, 337–342. doi: 10.1007/s40292-023-00584-3, PMID: 37261618 PMC10233551

[ref64] Spulber S. Conti M. Dupont C. Raciti M. Bose R. Onishchenko N.. (2015). Alterations in circadian entrainment precede the onset of depression-like behavior that does not respond to fluoxetine. Transl. Psychiatry 5:e603. doi: 10.1038/tp.2015.94, PMID: 26171984 PMC5068723

[ref65] Sun F. Liu M. Hu S. Xie R. Chen H. Sun Z.. (2024). Associations of weight-adjusted-waist index and depression with secondary infertility. Front. Endocrinol. 15:1330206. doi: 10.3389/fendo.2024.1330206, PMID: 38516413 PMC10956697

[ref66] Taillard J. Sagaspe P. Philip P. Bioulac S. (2021). Sleep timing, chronotype and social jetlag: impact on cognitive abilities and psychiatric disorders. Biochem. Pharmacol. 191:114438. doi: 10.1016/j.bcp.2021.114438, PMID: 33545116

[ref67] Tamrakar S. R. Bastakoti R. (2019). Determinants of infertility in couples. J Nepal Health Res Counc 17, 85–89. doi: 10.33314/jnhrc.1827, PMID: 31110383

[ref68] Textor J. Van Der Zander B. Gilthorpe M. S. Liskiewicz M. Ellison G. T. (2016). Robust causal inference using directed acyclic graphs: the R package 'dagitty'. Int. J. Epidemiol. 45, 1887–1894. doi: 10.1093/ije/dyw341, PMID: 28089956

[ref69] Toenders Y. J. Schmaal L. Nawijn L. Han L. K. M. Binnewies J. Van Der Wee N. J. A.. (2022). The association between clinical and biological characteristics of depression and structural brain alterations. J. Affect. Disord. 312, 268–274. doi: 10.1016/j.jad.2022.06.056, PMID: 35760189

[ref70] Tonon A. C. Carissimi A. Schimitt R. L. De Lima L. S. Pereira F. D. S. Hidalgo M. P. (2020). How do stress, sleep quality, and chronotype associate with clinically significant depressive symptoms? A study of young male military recruits in compulsory service. Braz. J. Psychiatry 42, 54–62. doi: 10.1590/1516-4446-2018-0286, PMID: 31166545 PMC6986495

[ref71] Uygur H. Tekdemir R. Uygur O. F. Aydin E. F. Celik M. Babacan H. E.. (2024). Psychometric properties of the Turkish reduced morningness and eveningness questionnaire. Chronobiol. Int. 41, 632–646. doi: 10.1080/07420528.2024.2339964, PMID: 38629999

[ref72] Uygur O. F. Uygur H. Chung S. Ahmed O. Demiroz D. Aydin E. F.. (2022). Validity and reliability of the Turkish version of the Glasgow sleep effort scale. Sleep Med. 98, 144–151. doi: 10.1016/j.sleep.2022.06.022, PMID: 35853331

[ref73] Vagos P. Rodrigues P. F. S. Pandeirada J. N. S. Kasaeian A. Weidenauer C. Silva C. F.. (2019). Factorial structure of the Morningness-Eveningness-stability-scale (MESSi) and sex and age invariance. Front. Psychol. 10:3. doi: 10.3389/fpsyg.2019.00003, PMID: 30705648 PMC6344426

[ref74] Van Den Berg J. F. Kivelä L. Antypa N. (2018). Chronotype and depressive symptoms in students: an investigation of possible mechanisms. Chronobiol. Int. 35, 1248–1261. doi: 10.1080/07420528.2018.1470531, PMID: 29764217

[ref75] Vo T. M. Tran Q. T. Le C. V. Do T. T. Le T. M. (2019). Depression and associated factors among infertile women at Tu Du hospital, Vietnam: a cross-sectional study. Int. J. Women's Health 11, 343–351. doi: 10.2147/ijwh.S205231, PMID: 31239787 PMC6551559

[ref76] Wang W. Yang F. Bai Y. Lu Y. Mao X. (2024). Association between domain-specific physical activity and mental health status after embryo transfer in IVF-ET-assisted pregnancy patients. Sci. Rep. 14:4928. doi: 10.1038/s41598-024-55097-3, PMID: 38418518 PMC10902343

[ref77] Wittmann M. Dinich J. Merrow M. Roenneberg T. (2006). Social jetlag: misalignment of biological and social time. Chronobiol. Int. 23, 497–509. doi: 10.1080/07420520500545979, PMID: 16687322

[ref78] Yang W. S. Fu W. X. Wang X. Deng Q. Wang L. Wang L. Y.. (2017). Comprehensive assessments of long-term sleep habits in epidemiological study: validity and reliability of sleep factors questionnaire (SFQ) among Chinese women. J. Psychosom. Res. 95, 12–18. doi: 10.1016/j.jpsychores.2017.02.005, PMID: 28314544

[ref79] Yao Q. Y. Yuan X. Q. Liu C. Du Y. Y. Yao Y. C. Wu L. J.. (2022). Associations of sleep characteristics with outcomes of IVF/ICSI treatment: a prospective cohort study. Hum. Reprod. 37, 1297–1310. doi: 10.1093/humrep/deac040, PMID: 35259255

[ref80] Yin L. Teklu S. Pham H. Li R. Tahir P. Garcia M. E. (2022). Validity of the Chinese language patient health questionnaire 2 and 9: a systematic review. Health Equity 6, 574–594. doi: 10.1089/heq.2022.0030, PMID: 36081885 PMC9448521

[ref81] Zegers-Hochschild F. Adamson G. D. Dyer S. Racowsky C. De Mouzon J. Sokol R.. (2017). The international glossary on infertility and fertility care, 2017. Hum. Reprod. 32, 1786–1801. doi: 10.1093/humrep/dex234, PMID: 29117321 PMC5850297

[ref82] Zhou Y. Sun Z. Song J. (2023). Research progress on the impact of anxiety and depression on embryo transfer outcomes of in vitro fertilization. Zhejiang Da Xue Xue Bao Yi Xue Ban 52, 61–67. doi: 10.3724/zdxbyxb-2022-0473, PMID: 37283119 PMC10293778

[ref83] Zhou Z. Zheng D. Wu H. Li R. Xu S. Kang Y.. (2018). Epidemiology of infertility in China: a population-based study. BJOG 125, 432–441. doi: 10.1111/1471-0528.14966, PMID: 29030908

[ref84] Zou H. Zhou H. Yan R. Yao Z. Lu Q. (2022). Chronotype, circadian rhythm, and psychiatric disorders: recent evidence and potential mechanisms. Front. Neurosci. 16:811771. doi: 10.3389/fnins.2022.811771, PMID: 36033630 PMC9399511

