# Supplementary material for: Association of chronotype and depression symptoms in Chinese infertile population undergoing assisted reproductive technology
Source: Front Psychol. 2025 Jun 13;16:1423418. doi: 10.3389/fpsyg.2025.1423418 (PMC12202667; doi:10.3389/fpsyg.2025.1423418)
Supplement: Supplementary file 1 [file Data_Sheet_1.zip › Supplemental Materials/Figure S1.docx]

**Figure S1.** Flow chart of selection of participants in the analysis.

Assessed for eligibility

*N* = 1243

*N* = 1066

Individuals were excluded if they

- Missing or duplicate data: n = 44

Final sample

*N* = 1022

Individuals were excluded

- Not meeting inclusion criteria or

declining to participate: n = 177
