# Supplementary material for: Association of chronotype and depression symptoms in Chinese infertile population undergoing assisted reproductive technology
Source: Front Psychol. 2025 Jun 13;16:1423418. doi: 10.3389/fpsyg.2025.1423418 (PMC12202667; doi:10.3389/fpsyg.2025.1423418)
Supplement: Supplementary file 1 [file Data_Sheet_1.zip › Supplemental Materials/Figure S3.docx]

A_m_

A_f_

P_fm_

Female’s

Chronotype

Male’s

Depression symptoms

**Figure S3.** Actor–Partner Interdependence Model of chronotype and depression symptoms in infertile couples.

Note: A_m_: actor effect of male’s chronotype on their depression symptoms; A_f_: actor effect of female’s chronotype on their depression symptoms; P_fm_: partner effect of female’s chronotype on male’s depression symptoms; P_mf_: partner effect of male’s chronotype on female’s depression symptoms; E_m_ and E_f_: residual errors on depression symptoms for male and female, respectively.

Male’s

Chronotype

Female’s

Depression symptoms

P_mf_
