# Supplementary material for: Association of chronotype and depression symptoms in Chinese infertile population undergoing assisted reproductive technology
Source: Front Psychol. 2025 Jun 13;16:1423418. doi: 10.3389/fpsyg.2025.1423418 (PMC12202667; doi:10.3389/fpsyg.2025.1423418)
Supplement: Supplementary file 1 [file Data_Sheet_1.zip › Supplemental Materials/Table S1.docx]

**Table S1.** The characteristics of participants according to depression symptoms phenotypes ^†^.

| Characteristic | Overall | Depression symptoms phenotypes ^‡^ | | *P* |
| --- | --- | --- | --- | --- |
|  |  | non-Depression | Depression |  |
| No. of Participants | 1022 | 495(48.4) | 527(51.6) |  |
| Age (%) |  |  |  | 0.002 |
| ≤29 | 377 (36.9) | 172 (34.7) | 205 (38.9) |  |
| 30-34 | 457 (44.7) | 219 (44.2) | 238 (45.2) |  |
| ≥35 | 188 (18.4) | 104 (21.0) | 84 (15.9) |  |
| Sex (Female, %) | 608 (59.5) | 270 (54.5) | 338 (64.1) | 0.090 |
| Education (%) |  |  |  | 0.557 |
| Middle school or below | 302 (29.5) | 152 (30.7) | 150 (28.5) |  |
| High/ vocational school | 382 (37.4) | 177 (35.8) | 205 (38.9) |  |
| College degree or above | 338 (33.1) | 166 (33.5) | 172 (32.6) |  |
| Annual incomes (%) |  |  |  | 0.086 |
| <30, 000 | 388 (38.0) | 185 (37.4) | 203 (38.5) |  |
| 30, 000-60, 000 | 242 (23.7) | 105 (21.2) | 137 (26.0) |  |
| ≥60, 000 | 392 (38.4) | 205 (41.4) | 187 (35.5) |  |
| Passive smoking (%) |  |  |  | 0.172 |
| Never | 326 (31.9) | 144 (29.1) | 182 (34.5) |  |
| Occasionally | 591 (57.8) | 299 (60.4) | 292 (55.4) |  |
| Frequently | 105 (10.3) | 52 (10.5) | 53 (10.1) |  |
| Physical activity (%) |  |  |  | 0.343 |
| Low | 342 (33.5) | 165 (33.3) | 177 (33.6) |  |
| Moderate | 341 (33.4) | 175 (35.4) | 166 (31.5) |  |
| Vigorous | 339 (33.2) | 155 (31.3) | 184 (34.9) |  |
| Cause of infertility (%) |  |  |  | 0.362 |
| Male factor | 179 (17.5) | 84 (17.0) | 95 (18.0) |  |
| Female factor | 365 (35.7) | 180 (36.4) | 185 (35.1) |  |
| Both | 249 (24.4) | 111 (22.4) | 138 (26.2) |  |
| Unexplained | 229 (22.4) | 120 (24.2) | 109 (20.7) |  |
| Living children (yes, %) | 162 (15.9) | 92 (18.6) | 70 (13.3) | 0.025 |
| Infertility treatment time |  |  |  | 0.057 |
| ≤6 month | 342 (33.5) | 182 (36.8) | 160 (30.4) |  |
| 7-12 month | 217 (21.2) | 107 (21.6) | 110 (20.9) |  |
| 13-24 month | 310 (30.3) | 144 (29.1) | 166 (31.5) |  |
| >24 month | 153 (15.0) | 62 (12.5) | 91 (17.3) |  |
| Frequency of insomnia (%) |  |  |  | <0.001 |
| Never | 236 (23.1) | 161 (32.5) | 75 (14.2) |  |
| Occasionally or ≤3 per month | 687 (67.2) | 313 (63.2) | 374 (71.0) |  |
| ≥4 per month | 99 (9.7) | 21 (4.2) | 78 (14.8) |  |
| Nocturnal wake frequency (%) |  |  |  | <0.001 |
| Never | 158 (15.5) | 101 (20.4) | 57 (10.8) |  |
| Occasionally | 471 (46.1) | 234 (47.3) | 237 (45.0) |  |
| ≥1 per night | 393 (38.5) | 160 (32.3) | 233 (44.2) |  |
| Daytime napping (%) |  |  |  | 0.985 |
| Never | 295 (28.9) | 144 (29.1) | 151 (28.7) |  |
| <1 | 525 (51.4) | 253 (51.1) | 272 (51.6) |  |
| ≥1 | 202 (19.8) | 98 (19.8) | 104 (19.7) |  |
| Social jetlag (≥1 h, %) | 211 (20.6) | 99 (20.0) | 112 (21.3) | 0.677 |
| Nighttime sleep duration (≥8 h, %) | 680 (66.5) | 338 (68.3) | 342 (64.9) | 0.280 |
| Chronotypes (yes, %) | 527 (51.6) | 68 (70.8) | 364 (52.2) | <0.001 |
| Evening Chronotype | 96 (9.4) | 92 (18.6) | 70 (13.3) |  |
| Intermediate Chronotype | 697 (68.2) | 92 (18.6) | 70 (13.3) |  |
| Morning Chronotype | 229 (22.4) | 92 (18.6) | 70 (13.3) |  |

Note: ^†^Values were presented as mean ± SD or percentages; ^‡^non-Depression: 0–4 score; Depression: 5–27 score.
