# Supplementary material for: Association of chronotype and depression symptoms in Chinese infertile population undergoing assisted reproductive technology
Source: Front Psychol. 2025 Jun 13;16:1423418. doi: 10.3389/fpsyg.2025.1423418 (PMC12202667; doi:10.3389/fpsyg.2025.1423418)
Supplement: Supplementary file 1 [file Data_Sheet_1.zip › Supplemental Materials/Table S2.docx]

**Table S2**. Sensitivity analysis of odds ratios and 95% confidence intervals for the association between chronotype and depression symptoms among individuals without night/shifts work in infertility populations.

| Depressive symptoms | Chronotypes^†^ | | | Per 1-SD | *P*_trend_^d^ |
| --- | --- | --- | --- | --- | --- |
|  | Evening chronotypes | Intermediate chronotype | Morning chronotype |  |  |
| No. of cases/participants | 59/85 | 341/648 | 85/215 |  |  |
| Model 1^a^ | Reference | 0.49 (0.30-0.80) ^⁎⁎^ | 0.29 (0.17-0.49) ^⁎⁎⁎^ | 0.67 (0.59-0.77) ^⁎⁎⁎^ | <0.001 |
| Model 2 ^b^ | Reference | 0.49 (0.30-0.82) ^⁎⁎^ | 0.31 (0.18-0.54) ^⁎⁎⁎^ | 0.66 (0.58-0.77) ^⁎⁎⁎^ | <0.001 |
| Model 3^c^ | Reference | 0.51 (0.30-0.87) ^⁎⁎^ | 0.32 (0.18-0.58) ^⁎⁎⁎^ | 0.67 (0.52-0.78) ^⁎⁎⁎^ | <0.001 |

Note: ^†^Evening Chronotype: 4–11 score; Intermediate Chronotype: 12–17 score; Morning Chronotype: 18–25 score. ^⁎^*P* <0.05, ^⁎⁎^*P* <0.01, ^⁎⁎⁎^ *P* <0.001. ^a^ Model 1: it did not adjust for the covariates. ^b^ Model 2: it additionally included age, sex, education, annual incomes, passive smoking, physical activity, cause of infertility, living children, and infertility treatment time. ^c^ Model 3: it additionally included frequency of insomnia, nocturnal wake frequency, daytime napping, social jetlag, and nighttime sleep duration. ^d^ The trend test was performed by assigning medians into three groups and using them as a continuous variable in the models.
