# Supplementary figures and images for: Association of chronotype and depression symptoms in Chinese infertile population undergoing assisted reproductive technology
Source: Front Psychol. 2025 Jun 13;16:1423418. doi: 10.3389/fpsyg.2025.1423418 (PMC12202667; doi:10.3389/fpsyg.2025.1423418)

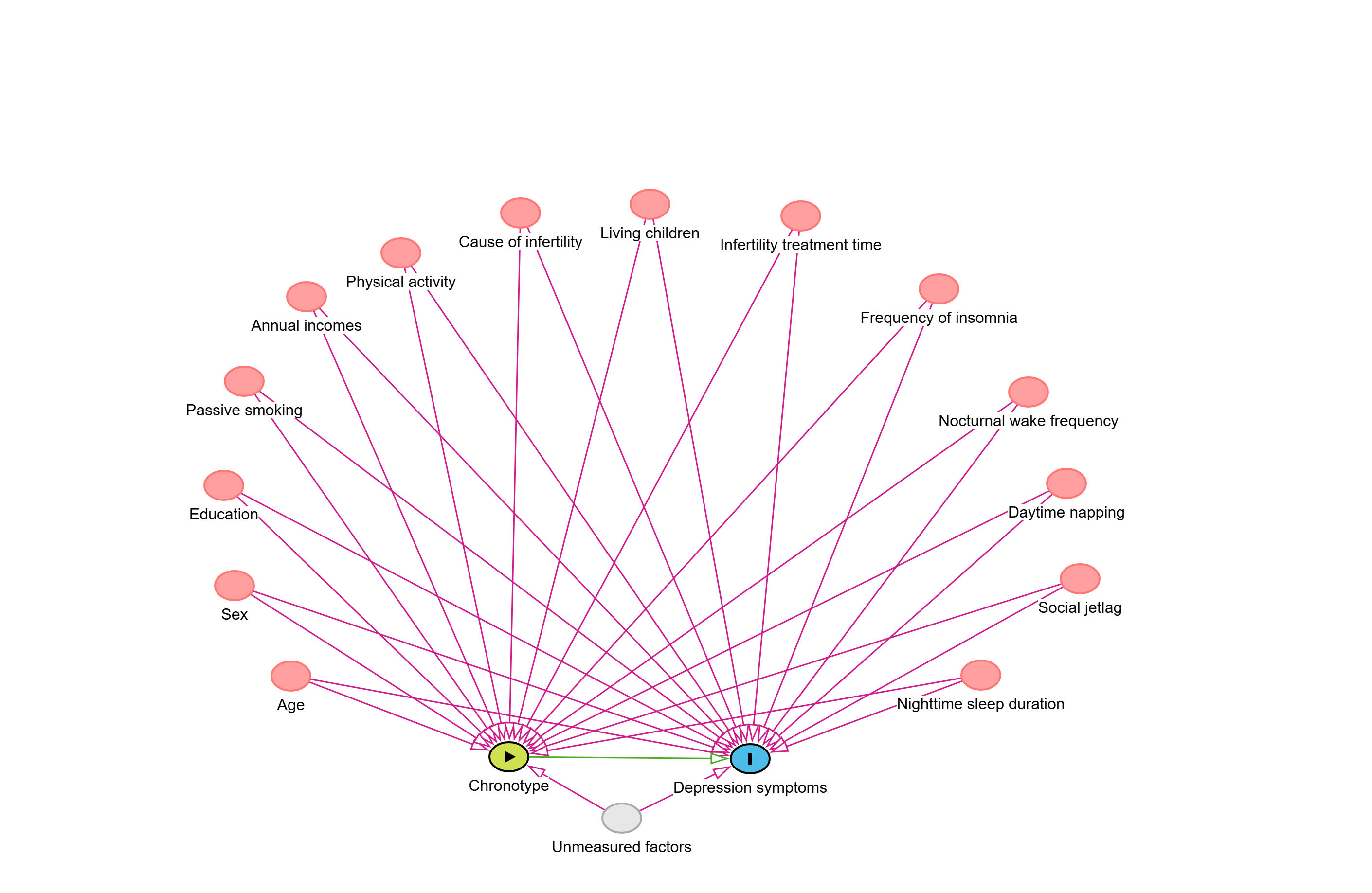

Supplement: Supplementary file 1 [file Data_Sheet_1.zip › Supplemental Materials/Figure S2.png]
